# Identification of *SERPINA1* promoting better prognosis in papillary thyroid carcinoma along with Hashimoto’s thyroiditis through WGCNA analysis

**DOI:** 10.3389/fendo.2023.1131078

**Published:** 2023-06-30

**Authors:** Yihan Zhang, Xin Xie, Hong Zhou, Bingxin Li, Li Ding, Zhaogen Cai, Huaidong Song, Shuangxia Zhao, Huanbai Xu

**Affiliations:** ^1^ Department of Endocrinology and Metabolism, Center for Microbiota and Immunological Diseases, Shanghai General Hospital, Shanghai Jiao Tong University School of Medicine, Shanghai, China; ^2^ Department of Endocrinology and Metabolism, Shanghai Traditional Chinese and Medicine Integrated Hospital, Shanghai, China; ^3^ Department of Pathology, The First Affiliated Hospital of Bengbu Medical College, Bengbu, Anhui, China; ^4^ Department of Molecular Diagnostics & Endocrinology, The Core Laboratory in Medical Center of Clinical Research, Shanghai Ninth People’s Hospital, Shanghai Jiao Tong University School of Medicine, Shanghai, China

**Keywords:** Hashimoto autoimmune thyroiditis, papillary thyroid cancer, weighted gene co- expression network analyses (WGCNA), SERPINA1, gene set enrichment analyses (GSEA)

## Abstract

**Background:**

Hashimoto’s thyroiditis (HT) is an autoimmune thyroid disease. Papillary thyroid carcinoma (PTC) is the most common endocrine cancer. In recent years the rate of coexistence between PTC and HT has increased but the relationship between them remains unclear, meaning it is necessary to find potential biomarkers for PTC coexistence with HT to predict its potential pathways.

**Method:**

A co-expression network was constructed using the weighted gene co-expression network analysis (WGCNA) in the R package. The modules of PTC associated with HT (PTC-W) were identified from the GSE138198 dataset. Protein-protein interaction network (PPI) was used to screen the hub genes. Immunohistochemical (IHC) analysis was performed to validate the expression of the hub genes in tissues. Clinical data from The Cancer Genome Atlas (TCGA) datasets were used to analyse the prognosis of the hub genes. Gene set enrichment analysis (GSEA) was used to screen potential pathways of PTC-W.

**Result:**

The MEbrown module representing the most significant module, with 958 differentially expressed genes (DEGs), was screened in PTC-W, based on WGCNA analysis. Through PPI, *SERPINA1* was identified as a hub gene. Immunostaining validated that *SERPINA1* was highly expressed in PTC-W. Moreover, PTC-W expressing *SERPINA1* exhibits a better prognosis than PTC without HT (PTC-WO).

**Conclusion:**

Our study demonstrates that *SERPINA1* promotes the occurrence of PTC-W, and its prognosis is better than PTC-WO. *SERPINA1* promotes a better prognosis for PTC-W, possibly through a tumour inhibition signalling pathway.

## 1 Introduction

Hashimoto’s thyroiditis (HT) is an autoimmune disease that affects the thyroid glands and is otherwise known as chronic lymphocytic or autoimmune thyroiditis. It is characterized by enlarged thyroid glands and parenchymal lymphocytic infiltration, with specific antibodies against the thyroid glands (1). HT, along with Graves’ disease, is considered to be an autoimmune thyroid disorder, and its incidence has increased significantly in recent years (2). The general prevalence of HT is around 10–12% and its incidence increases every year (1). Moreover, 20% patients of with autoimmune thyroid disorder present with complications involving other organ-specific/systemic autoimmune disorders (3).

Thyroid carcinoma, the most common carcinoma affecting the endocrine system, is an epithelial tumour of the thyroid gland. Papillary thyroid carcinoma (PTC), the largest type of thyroid cancer, accounts for approximately 80% of all thyroid cancers (4).

Epidemiologic studies have reported that the mean coexistence rate between HT and PTC is high, approximately 23% (range: 5%-85%), and has increased recently (5). Furthermore, HT is considered to potentially affect the development and progression of PTC. The prevalence and risk ratio of PTC is significantly higher in HT patients than in non-HT patients (6), meaning HT may be a potential risk factor for PTC. However, the specific association between HT and PTC remains unclear and controversial (7). Fine needle Aspiration Biopsy (FNAB) studies based on populations report the absence of a specific connection between HT and PTC, whereas many studies on thyroid postoperative specimens have reported a positive correlation between them (7). Moreover, the prognosis of PTC associated with HT is still controversial (8, 9). Some studies have found that PTC associated with HT (PTC-W) exhibited better clinical behavioural characteristics and prognoses than PTC without HT (PTC-WO) (8) but other studies did not provide similar conclusions (9, 10). PTC associated with HT is often observed with reactive hyperplasia in central lymph nodes (11) and previous studies have speculated that the mechanism involves HT being associated with lymphocyte infiltration and facilitating antitumour immunity in PTC (12).

Bioinformatics analysis is a method of analyzing biological information by computer that has become an indispensable part of the biomedical field. Not only can it be used to study genetics, but also proteomics, metabolomics, microbiome, and so on (13). Next-generation sequencing (NGS), a recently high-throughput sequencing technology of bioinformatics analysis growing in popularity, can deeply sequence target regions (14), which provides us with a new perspective on the genome and the disease. These technologies can potentially find biomarkers for PTC-W to predict its potential pathways through bioinformatics analysis.

## 2 Materials and methods

### 2.1 Data sources

**Figure 1:**
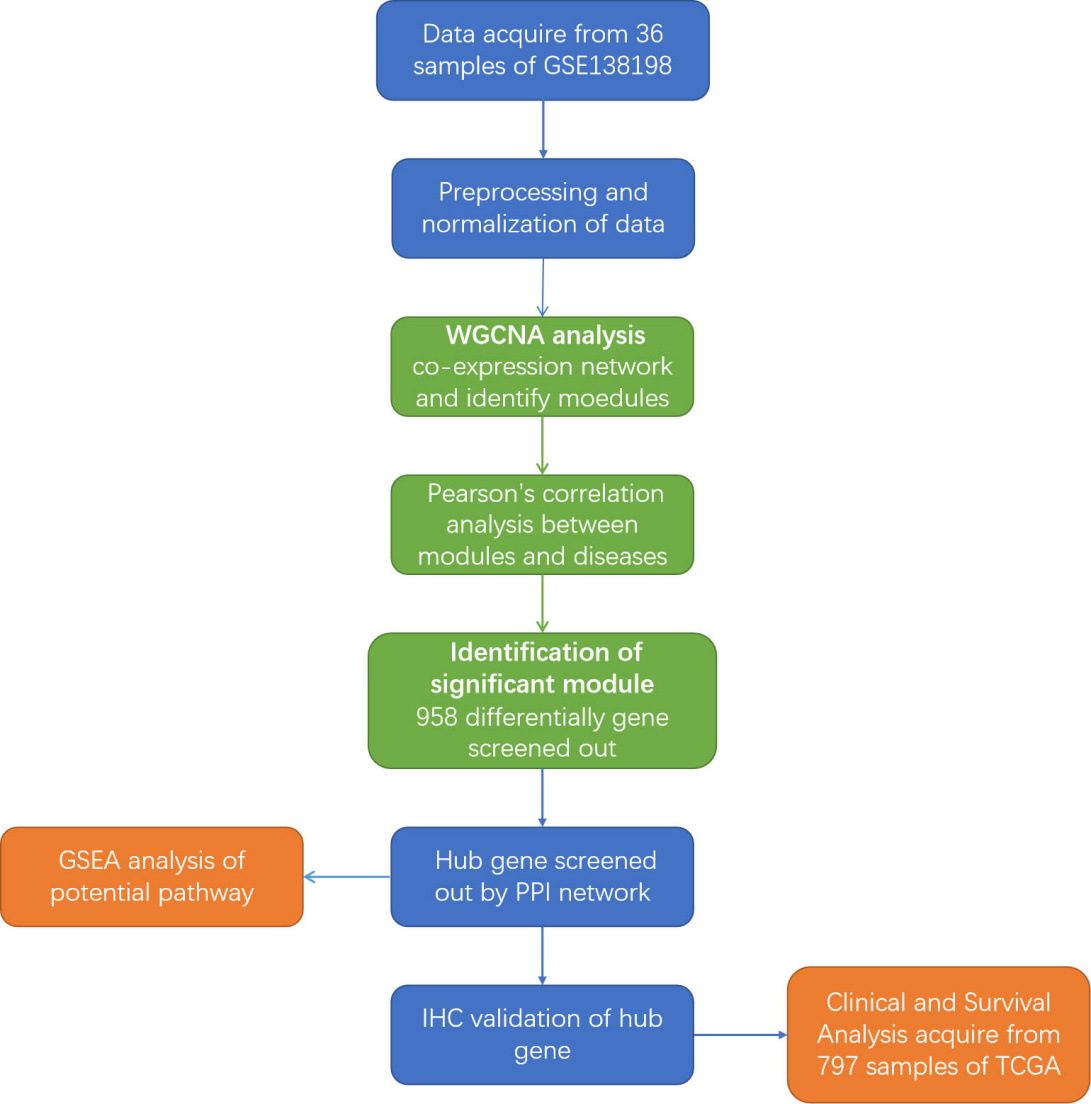
Flowchart of the study. GEO, Gene Expression Omnibus; WGCNA, weighted gene co-expression network; GSEA, Gene Set Enrichment Analysis; IHC, Immunohistochemistry; PPI, Protein-protein interaction; TCGA, The Cancer Genome Atlas.

The Gene Expression Omnibus (GEO) database, a high-throughput functional genomic database, was used for data analysis. For the functional genomics analysis, we searched the next-generation sequencing data in the GEO database. A flowchart of the entire study is shown in **Figure 1**. The criteria for data screening were as follows: (1) keywords ‘PTC and HT’ were used to search for datasets; (2) the dataset must include PTC-W, PTC-WO, HT, and normal samples; (3) the sample size was greater than 30; and (4) that the dataset used gene expression chips. GSE138198 (15), with 36 samples (13 HT, 8 PTC-W, 6 PTC-WO, and 3 normal (NOR)), was selected for the subsequent analysis, which aimed to detect the DEGs involved in PTC-W.

### 2.2 Original data analysis

The original data from the GSE138198 dataset were downloaded from the GEO database. The limma package for R (version 4.1) was used to analyse the data, with the following criteria: false discovery rate < 0.05 and a log2 fold change ≥ 0.5. The data were normalized using the robust multi-array average (RMA) package.

### 2.3 Module and DEG identification based on weighted gene co-expression network analysis

A co-expression network was constructed using the WGCNA package from R. First, the samples were clustered to assess the presence of significant outliers. Next, the automatic network constructor was used to develop the co-expression network. The soft thresholding power β was calculated using the pickSoft threshold function in R, and the common expression similarity was improved to calculate the adjacency degree. Then, modules were detected through hierarchical clustering and by using the dynamic tree-cutting function. Finally, we calculated the gene significance and module membership of the modules correlated with the disease groups. The gene information of the corresponding module was included in the next analysis. Finally, we visualized the characteristic gene network. The genes with a gene significance (GS) of greater than 0.2 and module membership (MM) greater than 0.8 in the most significant gene module network were defined as DEGs.

### 2.4 Protein-protein interaction network construction

To further detect the physical and functional interactions of hub genes at the protein level, a PPI network was constructed using the STRING database (http://string-db.org, version 10). All hub genes were imported to the STRING database, and medium confidence (0.400) was set for the interaction score. The analysis results were downloaded and imported to Cytoscape, version 3.8.0. Then, the network data was analysed using the MCODE plugin in Cytoscape to construct the cluster gene modules using the k-core algorithm, which reflected the importance of each gene.

### 2.5 Immunohistochemistry

Paraffin-embedded thyroid tissue specimens from 10 patients with PTC-W, 10 patients with PTC-WO, 10 patients with HT, and 10 normal people were collected from the Shanghai General Hospital, from September 2020 to May 2022. We collected PTC and HT tissues from patients with PTC-W, which include papillary carcinoma cells and lymphocyte infiltrated tissues. Subsequently, three comparison groups were defined: PTC-W vs. NOR; PTC-WO vs. NOR; and, HT vs. NOR.

The samples were incubated with the rabbit monoclonal anti-serpin family A member 1 (*SERPINA1*) antibody (1:100 dilution; Cat. ab220161; Abcam, USA) overnight, at 4 °C. In the semiquantitative process, the Image-Pro Plus software (version 6.0) was used to assess the area and mean densitometry (magnification: ×200) of the stained area, as well as the integrated optical density values of the IHC sections.

All experimental procedures were approved by the Ethical Committee of the SHANGHAI General Hospital. Signed written informed consent was obtained from all patients.

### 2.6 Clinical and survival analysis

The clinical data for PTC-W and PTC-WO were downloaded from The Cancer Genome Atlas database (https://tcga-data.nci.nih.gov/tcga/). A total of 734 patients with PTC-WO and 63 patients with PTC-W were included in this analysis. We compared several pivotal clinical traits such as age, gender, and TNM stage between the PTC-WO and PTC-W groups, to see if there were significant differences. The prognostic date of the hub genes was analysed *via* the Kaplan-Meier survival analysis and log-rank test. The group was divided into a high expression group and a low expression group of the hub gene. Survival curves were constructed using the “survival” package in R, and *p* < 0.05 was considered statistically significant.

### 2.7 Statistical analysis

All statistical analyses were performed using R, version 3.6.1. The clinical data have been presented as mean ± standard deviation. Data with *p* < 0.05 were considered statistically significant. SPSS Statistics (version 26.0) and GraphPad Prism (version 8.0) software were used to analyse the data. Chi-square and Fisher’s exact (when the number of patients < 5) tests were used to assess the association of each categorical variable. Two-sided Student’s t-tests were used for the significance test of the comparison between the two groups. Univariate and multivariate analyses of overall survival were performed using Cox regression, and hazard ratios were calculated using 95% CIs. All p-values were two-sided, and data with *p ≤*0.05 were considered statistically significant.

### 2.8 GSEA analysis

Gene set enrichment analysis (GSEA, version 4.0.3) was used to analyse the potential pathways through which PTC was associated with HT. GSEA was performed on hallmark gene sets in groups with high hub gene expression. Upregulated gene sets were selected as significant gene sets.

## 3 Results

### 3.1 Identification of DEGs in PTC-W based on WGCNA analysis

**Figure 2:**
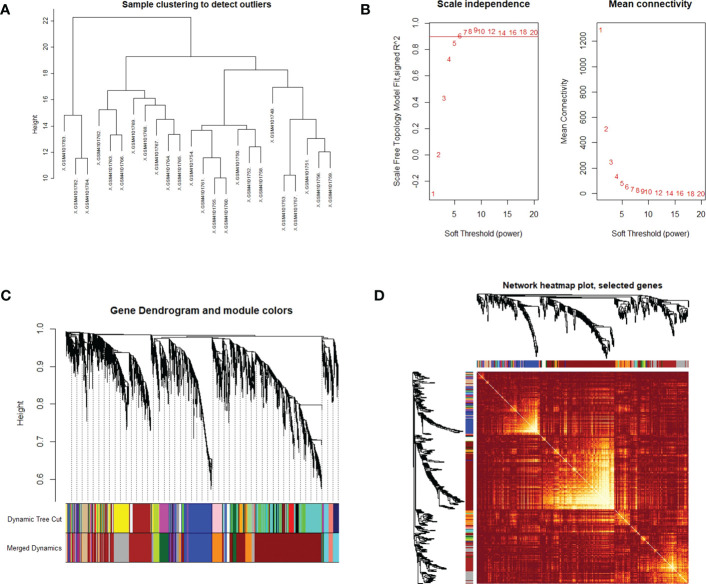
**(A)** Clustering dendrogram of the samples based on their Euclidean distance. The sample was taken from the GSE138198 dataset containing 21 Hashimoto’s thyroiditis (HT) and 3 normal samples, of the GEO database. **(B)** Analysis of network topology for various soft thresholding powers. The x-axis reflects the soft thresholding power. The y-axis reflects the scale-free topology model fit index. The soft thresholding power β was set at 6, with the scale independence reaching 0.9. **(C)** Clustering dendrogram of genes with dissimilarity, based on the topological overlap, together with assigned module colours. **(D)** Visualization of the WGCNA network using a heatmap plot. The heatmap depicts the topological overlap matrix (TOM) among all modules included in the analysis. The light colour represents an increasing overlap, and the progressively darker red colour represents a low overlap.

**Figure 3:**
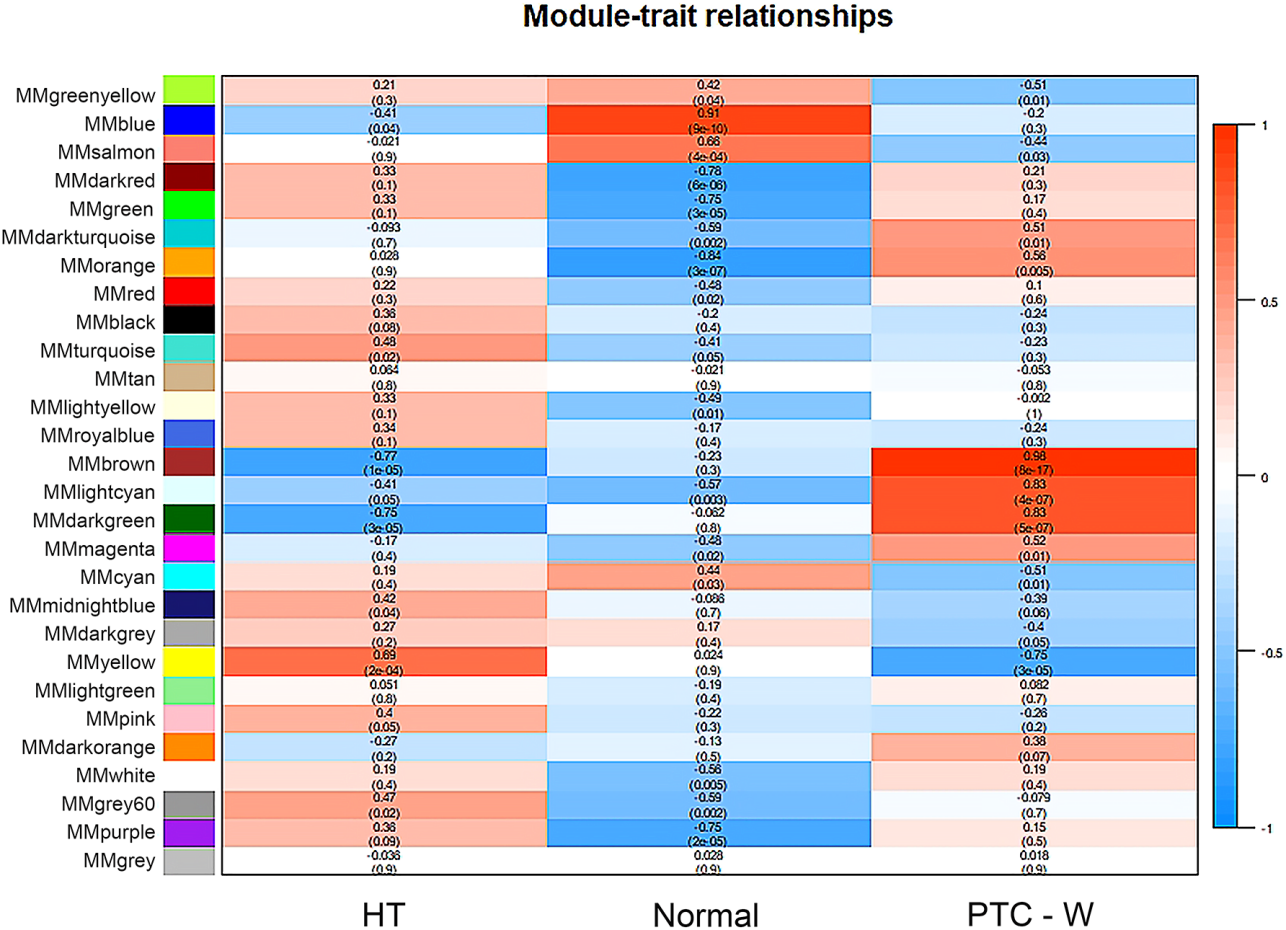
Module–trait associations. Each row corresponds to a module, and each column corresponds to a trait. Each cell contains the corresponding correlation and p-value. The table is colour-coded by correlation, according to the colour legend. To get an insight into the function of the DEGs of PTC-W, 958 DEGs were analysed in the MEbrown module.

WGCNA was used to identify highly related genes and construct a co-expression network, to clarify the central modules and genes in PTC-W. All samples were within the standards, and after quality assessment, all of them were included in the analysis (**Figure 2A**). The soft thresholding power β was set at 6, with the scale independence reaching 0.9 (**Figure 2B**). A cluster dendrogram of genes was constructed based on the dissimilarity of the topological overlaps, along with the specified module colours (**Figure 2C**). After analysing the interactions between gene modules, the topological overlap matrix diagrams of a gene network were generated based on the corresponding hierarchical cluster dendrograms and modules (**Figure 2D**). Finally, 28 gene modules were screened using the hierarchical clustering dendrogram, listed in **Figure 3**. Each row represents a module, and each column represents a disease. Each cell indicates the correlation and *p*-value. According to the colour legend, the table is colour-coded according to the correlation. Consequently, the MEbrown module represents the most significant module, with 958 DEGs (*p* = 8E-17).

### 3.2 Identification of hub gene related to PTC-W

**Figure 4:**
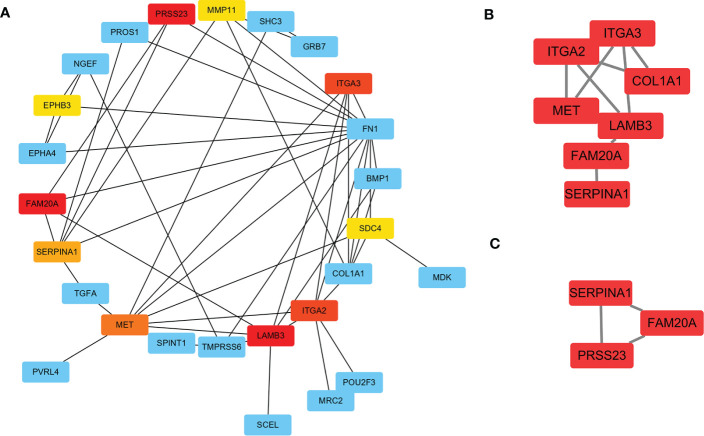
Construction of PPI network for DEGs using STRING. **(A)** The significant module consists of 23 nodes and 48 edges of the PPI network. Nodes represent the genes and edges represent the protein interaction between genes. The colour of nodes represents the degree of association between the proteins. Red means high association, and yellow means low association. **(B)** Cluster genes *via* MCODE (Score: 3.4) **(C)** Cluster genes *via* MCODE (Score: 3.0). Combining the results of A–C, *SERPINA1*, and *FAM20A* were highly correlated as hub genes.

To further investigate the DEGs and the underlying protein levels, we constructed a PPI network using the STRING database. As shown in **Figure 4A**, the core PPI network includes 23 nodes and 48 edges. Next, the network data was analysed using MCODE to construct the cluster gene modules with k-core, which reflected the importance of each gene. The genes in module A that were clustered by MCODE, were listed in module B (Score: 3.4), showing in **Figure 4B**. To filter further, we built a smaller module C (Score: 3.0), as shown in **Figure 4C**. Finally, the common genes in both modules were listed as hub genes. Consequently, two genes were selected as hub genes: *SERPINA1* and *FAM20A*. To further screen the hub gene, we compared the expression level of these two genes from GEO datasets through WGCNA analysis. We found that the GS and MM of *SERPINA1* were 0.86 and 0.85, and the GS and MM of *FAM20A* were 0.81 and 0.80. Based on these findings, *SERPINA1* was selected as the hub gene of PTC-W for further study.

### 3.3 Validation of *SERPINA1* up-expressed in PTC-W

**Figure 5:**
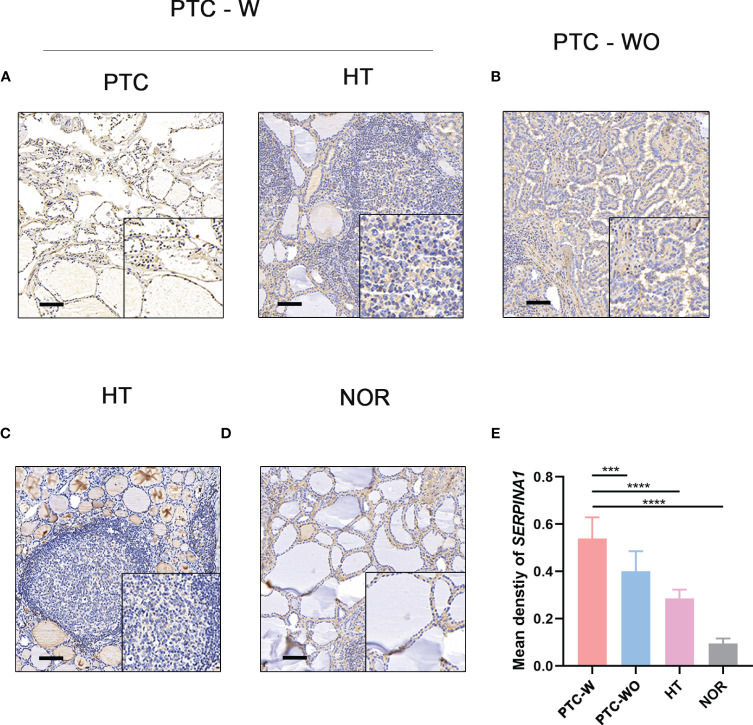
IHC of *SERPINA1* in tissues. *SERPINA1* was upregulated in PTC-W, compared to PTC-WO and NOR. **(A)** IHC of *SERPINA1* in PTC-W. **(B)** IHC of *SERPINA1* in PTC-WO. **(C)** IHC of *SERPINA1* in HT. **(D)** IHC of *SERPINA1* in NOR. The original magnification was 200× and the lower upper quadrant was 400×. The scale bar is 50 mm. **(E)** The mean density of *SERPINA1* in PTC-W, PTC-WO, HT, and NOR groups of the semiquantitative process. A significant difference was detected between these groups (***p < 0.001, ****p < 0.0001).

To validate the selected hub gene, the IHC of hub genes were used to validate them in PTC-W. *SERPINA1* was more expressed in PTC-W than in PTC-WO, HT, and NOR (**Figures 5A-D**). Meanwhile, semiquantitative IHC also showed that the expression of *SERPINA1* gradually increased in PTC-W, PTC-WO, HT, and NOR (**Figure 5E**). This means that *SERPINA1* may promote the occurrence of PTC with HT. Moreover, it also suggests that patients with HT who express *SERPINA1* are more likely to develop PTC.

### 3.4 The PTC-W group had a better prognosis than the PTC-WO group

**Figure 6:**
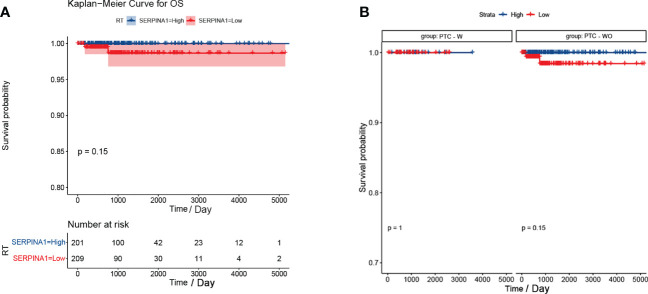
Kaplan-Meier curve of *SERPINA1*. **(A)** Kaplan-Meier curve analysis between *SERPINA1* high-group and *SERPINA1* low-group. **(B)** *SERPINA1* - stratified subgroup analysis was performed for overall survival between the PTC-W and PTC-WO groups.

Our analysis found that there was no difference between the PTC-W and PTC-WO groups in terms of gender, stage, tumour node, metastasis, and age (**Table 1**). However, there was a difference between the PTC-W and PTC-WO groups in terms of the node (*p* = 0.038). The univariate COX analysis shows that age, stage II, III, IV, IVA, and IVC were the risk factors (**Table 2**). In multivariate analysis, age, stage, T4, and M1 were independent risk factors for PTC-W. Moreover, the results of KM curves demonstrated that the high expression of *SERPINA1* correlated to a better prognosis, as shown in **Figure 6A**. Likewise, the Kaplan–Meier curves subgroup analysis of *SERPINA1* showed that the PTC-WO group exhibited lower overall survival, compared to the PTC-W group, as shown in **Figure 6B**. However, no statistical significance was observed. We speculate that the reasons for this might be: 1) the time of follow-up was not long enough for the positive events to occur; and/or 2) as the diagnosis and treatment are now near instantaneous, the probability of positive events is relatively low.

**Table 1:** Univariate analysis of PTC-W and PTC-WO.

Characteristic	PTC-W	PTC-WO	p
n	63	734	
gender, n (%)			0.577
female	49 (6.1%)	541 (67.9%)	
male	14 (1.8%)	193 (24.2%)	
stage, n (%)			0.808
Stage I	43 (5.4%)	437 (54.8%)	
Stage II	5 (0.6%)	65 (8.2%)	
Stage III	11 (1.4%)	148 (18.6%)	
Stage IV	0 (0%)	2 (0.3%)	
Stage IVA	4 (0.5%)	66 (8.3%)	
Stage IVB	0 (0%)	1 (0.1%)	
Stage IVC	0 (0%)	15 (1.9%)	
T, n (%)			0.203
T1	7 (1.3%)	63 (11.4%)	
T1a	0 (0%)	20 (3.6%)	
T1b	12 (2.2%)	67 (12.1%)	
T2	26 (4.7%)	149 (26.9%)	
T3	18 (3.2%)	166 (30%)	
T4	0 (0%)	10 (1.8%)	
T4a	0 (0%)	14 (2.5%)	
TX	0 (0%)	2 (0.4%)	
M, n (%)			0.083
M0	31 (5.6%)	301 (54.3%)	
M1	0 (0%)	9 (1.6%)	
MX	32 (5.8%)	181 (32.7%)	
N, n (%)			**0.038**
N0	29 (5.2%)	209 (37.7%)	
N1	5 (0.9%)	95 (17.1%)	
N1a	17 (3.1%)	75 (13.5%)	
N1b	9 (1.6%)	66 (11.9%)	
NX	3 (0.5%)	46 (8.3%)	
age, median (IQR)	44 (35, 55)	44 (33, 55.71)	0.563

Bold values means significant difference, that is, p value < 0.05.

**Table 2:** Risk factors based on univariate analyses and multivariate COX analyses.

Characteristics	Total(N)	Univariate analysis		Multivariate analysis	
		Hazard ratio (95% CI)	*P* value	Hazard ratio (95% CI)	*P* value
age	797	1.137 (1.096-1.178)	**<0.001**	1.170 (1.111-1.232)	**<0.001**
Gender					
male	207	Reference			
female	590	0.468 (0.209-1.047)	0.064	0.758 (0.272-2.116)	0.597
stage	797				
Stage I	480	Reference			
Stage II	70	6.107 (1.226-30.425)	**0.027**	0.535 (0.152-1.884)	0.331
Stage III	159	9.489 (2.565-35.105)	**<0.001**	0.315 (0.119-0.831)	**0.020**
Stage IV	2	149.583 (15.122-1479.623)	**<0.001**	0.218 (0.028-1.717)	0.148
Stage IVA	70	11.226 (2.505-50.315)	**0.002**	0.074 (0.017-0.329)	**<0.001**
Stage IVB	1	0.000 (0.000-Inf)	0.998	1.000 (1.000-1.000)	
Stage IVC	15	39.285 (9.329-165.430)	**<0.001**	868509.880 (188704.265-3997309.832)	**<0.001**
T	554				
T1	70	Reference			
T2	175	0.900 (0.165-4.916)	0.903	1.977 (0.641-6.093)	0.235
T3	184	1.082 (0.210-5.583)	0.925	1.917 (0.674-5.454)	0.223
T4	10	10.661 (1.944-58.459)	**0.006**	13.670 (4.399-42.481)	**<0.001**
T4a	14	5.292 (0.743-37.704)	0.096	12.343 (2.764-55.114)	**<0.001**
TX	2	0.000 (0.000-Inf)	1.000	0.000 (0.000-Inf)	1.000
M	554				
M0	332	Reference			
M1	9	5.181 (1.128-23.797)	**0.034**	0.000 (0.000-0.000)	**<0.001**
MX	213	0.725 (0.247-2.130)	0.559	0.760 (0.261-2.217)	0.615
N	554				
N0	238	Reference			
N1	100	0.876 (0.218-3.517)	0.852		
N1a	92	0.717 (0.144-3.555)	0.684		
N1b	75	2.137 (0.532-8.585)	0.285		
NX	49	2.156 (0.537-8.649)	0.279		

Bold values means significant difference, that is, p value < 0.05.

### 3.5 *SERPINA1* was associated with tumour inhibition signalling pathways in PTC-W

**Figure 7:**
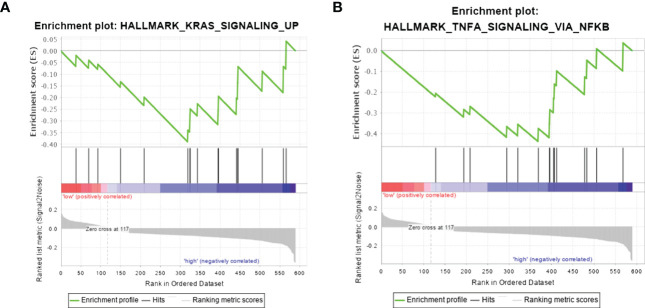
Gene set enrichment analysis in samples exhibiting high expression of *SERPINA1*. Hallmark pathway gene sets were used to characterise the potential signalling pathways in PTC-W. **(A)** High expression of *SERPINA1* inhibits the KRAS signalling pathway. **(B)** High expression of *SERPINA1* inhibits TNF-α signalling *via* NF-kB.

GSEA was performed to find the potential pathway of *SERPINA1* in PTC-W, as illustrated in **Figure 7**. The results showed that high expression of *SERPINA1* inhibits the Kirsten rat sarcoma 2 viral oncogene homolog (KRAS) signalling pathway (**Figure 7A**) and TNF-α signalling *via* NF-kB (**Figure 7B**). As a result, we assume that the PTC-W group exhibiting *SERPINA1* expression has a better prognosis than the PTC-WO group exhibiting *SERPINA1* expression, possibly because it involved tumour inhibition signalling pathways.

## 4 Discussion

HT is an autoimmune thyroid disease whose morbidity increases significantly every year. Hence, it is associated with many other organ-specific/systemic autoimmune disorders. Previous studies have demonstrated a significant association between autoimmune thyroiditis and the following diseases: chronic autoimmune gastritis (CAG), vitiligo (Vit), rheumatoid arthritis, polymialgia rheumatica (Polym), celiac disease, diabetes, sjogren disease, multiple sclerosis, systemic lupus erythematosus, sarcoidosis, alopecia, psoriathic arthritis, systemic sclerosis, HCV-related cryoglobulinemia, polymyalgia rheumatica, diabetes (type 1), and Sjogren disease (16, 17). As for the potential mechanism, another study has mentioned that Th1 lymphocytes may involve in the process of autoimmune response, with the expressions of the Th1 chemokine CXCL10 (18). However, the more specific mechanisms involved still need to be further explored and studied.

As a new data exploration tool and gene screening method, the function of WGCNA mainly includes the following aspects: 1) construction of the network and module; 2) significant module and gene mining; 3) topological calculation; and 4) data visualization (19). Compared to common gene screening methods, only WGCNA identifies the pattern of correlation among genes and reduces the false positive rate (20). We identified the most statistically significant modules (brown) in the PTC-W group, compared to the HT and NOR groups. The results demonstrated that hub genes within the above modules may serve as potential markers for PTC-W. When HT is associated with PTC, the hub genes are expressed in HT (within the infiltrating lymphocytes) specimens and papillary carcinoma cells, but not in normal thyroid specimens (21). This is consistent with our findings, further illustrating the credibility of our findings.

*SERPINA1* is a member of the serpin superfamily, which encodes a serine protease inhibitor, and targets trypsin, elastase, plasmin, chymotrypsin, thrombin, and the plasminogen activator (22). Previous studies have reported its association with PTC (23). Moreover, it has been reported that alternative splicing events in *SERPINA1* may improve the sensitivity and specificity of the diagnostic biomarkers in PTC (24). However, there were no studies demonstrating that *SERPINA1* was associated with PTC-W.

We found that PTC-W had a higher survival rate than PTC alone, which was consistent with the results of most prior research. Another study demonstrated three different mechanisms between HT and PTC, namely tumour development through 1) TSH stimulation, 2) expression of certain proto-oncogenes, and 3) chemokines and other molecules produced by the lymphocytic infiltrate (11). The Cox analysis in this study showed that age, stage, tumour 4, and metastasis 1 were the risk factors for PTC-W. Therefore, patients with these risk factors may require aggressive treatment, while for those without these risk factors, low-intensity therapy may be sufficient.

*KRAS*, a proto-oncogene, encodes a small GTPase transductor protein called KRAS (25). KRAS signalling requires adequate KRAS translation, plasma membrane localization, and interaction with effector proteins before it can be activated (25). At present, there are no studies that prove the relationship between *SERPINA1* using the KRAS signalling pathway. Moreover, tumor necrosis factor (TNF) plays an important role in apoptosis, inflammation, and immunity, and plays a vital role in the pathogenesis of many diseases, including cancer (26). A previous study showed that *SERPINA1*, produced by SCC cells, is upregulated by TNF-α, and is dependent on p38 mitogen-activated protein kinase activity (27). However, studies have not demonstrated that *SERPINA1* is associated with TNF-α in PTC. Hence, this study is the first to prove that *SERPINA1* inhibits high expression of KRAS and TNF-α signalling *via* the NF-kB pathway, thereby inhibiting PTC.

## 5 Conclusion

In our study, 958 DEGs were mined based on WGCNA analysis in PTC-w, and the key gene *SERPINA1* was identified by PPI and IHC. The results of clinical data analysis showed that *SERPINA1* may be associated with the occurrence of PTC with HT. As a result, this study shows that *SERPINA1* promotes the occurrence of PTC-W and that its prognosis is better than PTC-WO. *SERPINA1* promotes a better prognosis of PTC-W possibly through tumour inhibition signalling pathways, such as the KRAS and NF-kB signalling pathways. Hence, our research finds a potential target for novel molecular therapy, and also for a prognostic judgment to predict the risk of disease. Furthermore, it could also be a predictor of disease occurrence.

## Data availability statement

The original contributions presented in the study are included in the article/**Supplementary Material**. Further inquiries can be directed to the corresponding authors.

## Ethics statement

The studies involving human participants were reviewed and approved by the ethical committee of shanghai general hospital (2019SQ002). The patients/participants provided their written informed consent to participate in this study. Written informed consent was obtained from the individual(s) for the publication of any potentially identifiable images or data included in this article.

## Author contributions

YZ, XX, and BL conceived and designed the study. LD, ZC, and HS acquired the data; SZ, HX, and HZ analysed and interpreted the data. YZ and XX wrote the paper. All authors contributed to the article and approved the submitted version.
